# Metabolomic studies in the inborn error of metabolism alkaptonuria reveal new biotransformations in tyrosine metabolism

**DOI:** 10.1016/j.gendis.2021.02.007

**Published:** 2021-02-22

**Authors:** Brendan P. Norman, Andrew S. Davison, Juliette H. Hughes, Hazel Sutherland, Peter JM. Wilson, Neil G. Berry, Andrew T. Hughes, Anna M. Milan, Jonathan C. Jarvis, Norman B. Roberts, Lakshminarayan R. Ranganath, George Bou-Gharios, James A. Gallagher

**Affiliations:** a Institute of Life Course and Medical Sciences, University of Liverpool, William Henry Duncan Building, 6 West Derby Street, Liverpool, L7 8TX, UK; b Department of Clinical Biochemistry & Metabolic Medicine, Liverpool Clinical Laboratories, Royal Liverpool University Hospital, Prescot Street, Liverpool, L7 8XP, UK; c School of Sport & Exercise Sciences, Liverpool John Moores University, Tom Reilly Building, Byrom Street, Liverpool, L3 3AF, UK; d Department of Chemistry, University of Liverpool, Crown Street, Liverpool, L69 7ZD, UK

**Keywords:** Alkaptonuria, Biotransformation, Metabolism, Metabolomics, Mice, AKU, alkaptonuria, HGD, homogentisate 1,2-dioxygenase, HGA, homogentisic acid, LC-QTOF-MS, liquid chromatography quadrupole time-of-flight mass spectrometry, MS/MS, tandem mass spectrometry, AMRT, accurate mass/retention time, PCA, principal component analysis, RT, retention time, FDR, false-discovery rate, FC, fold change, QC, quality control, CV, coefficient of variation, MSC, Molecular Structure Correlator, HPPD, hydroxyphenylpyruvic acid dioxygenase

## Abstract

Alkaptonuria (AKU) is an inherited disorder of tyrosine metabolism caused by lack of active enzyme homogentisate 1,2-dioxygenase (HGD). The primary consequence of HGD deficiency is increased circulating homogentisic acid (HGA), the main agent in the pathology of AKU disease. Here we report the first metabolomic analysis of AKU homozygous *Hgd* knockout (*Hgd*^−/−^) mice to model the wider metabolic effects of *Hgd* deletion and the implication for AKU in humans. Untargeted metabolic profiling was performed on urine from *Hgd*^−/−^ AKU (*n* = 15) and *Hgd*^+/−^ non-AKU control (*n* = 14) mice by liquid chromatography high-resolution time-of-flight mass spectrometry (Experiment 1). The metabolites showing alteration in *Hgd*^−/−^ were further investigated in AKU mice (*n* = 18) and patients from the UK National AKU Centre (*n* = 25) at baseline and after treatment with the HGA-lowering agent nitisinone (Experiment 2). A metabolic flux experiment was carried out after administration of ^13^C-labelled HGA to *Hgd*^−/−^(*n* = 4) and *Hgd*^+/−^(*n* = 4) mice (Experiment 3) to confirm direct association with HGA. *Hgd*^−/−^ mice showed the expected increase in HGA, together with unexpected alterations in tyrosine, purine and TCA-cycle pathways. Metabolites with the greatest abundance increases in *Hgd*^−/−^ were HGA and previously unreported sulfate and glucuronide HGA conjugates, these were decreased in mice and patients on nitisinone and shown to be products from HGA by the ^13^C-labelled HGA tracer. Our findings reveal that increased HGA in AKU undergoes further metabolism by mainly phase II biotransformations. The data advance our understanding of overall tyrosine metabolism, demonstrating how specific metabolic conditions can elucidate hitherto undiscovered pathways in biochemistry and metabolism.

## Introduction

Alkaptonuria (AKU) is a rare disorder of tyrosine metabolism caused by congenital lack of activity of the enzyme homogentisate 1,2-dioxygenase HGD (E.C.1.12.11.5).^1^ The biochemical consequence of HGD deficiency is increased homogentisic acid (HGA) in the circulation, the pathognomonic sign of the disease and central to its pathophysiological features.^2^ HGA has a high affinity for collagenous tissues, where its deposition produces striking pigmentation,^3^ a process called ochronosis. Cartilage of load-bearing joints is particularly susceptible to ochronosis. Presence of HGA-derived pigment in these joints alters the physicochemical properties of cartilage that support normal transmission of load and results in an inevitable and severe early-onset osteoarthropathy.^4^^,^^5^

Metabolomics has emerged as an invaluable approach for studying AKU. In our laboratory we have developed a targeted approach with specific mass spectrometric assays as an aid for diagnosis and monitoring of AKU.^6^^,^^7^ These assays offer precise quantification of tyrosine pathway metabolites including HGA in serum and urine. More recently, a strategy has been developed for profiling and chemical identification of up to 619 related and more general metabolites by high-resolution accurate mass and retention time using liquid chromatography quadrupole time-of-flight mass spectrometry (LC-QTOF-MS).^8^ Application of this technique to AKU serum^9^ and urine^8^ enabled the discovery of previously unknown metabolite and metabolic pathway alterations following treatment with the HGA-lowering agent nitisinone. Metabolic profiling therefore has potential in AKU as both a phenotyping and biomarker discovery tool. However, to our knowledge, untreated AKU has not been compared with non-AKU at the metabolome level before.

The investigations into such possible changes in the metabolome as a result of AKU will be greatly facilitated by use of an animal model of AKU developed in our laboratory by homozygous knockout of the *Hgd* gene (*Hgd*^−/−^) in mice. The genetically altered *Hgd*^−/−^ mouse recapitulates human AKU, with elevated plasma and urine HGA, development of ochronosis and its inhibition by nitisinone.^10^^,^^11^ It was important to establish the metabolic profile of this model, so we first compared the urinary profiles of AKU homozygous *Hgd* knockout mice (*Hgd*^−/−^) with non-AKU heterozygous knockout (*Hgd*^+/−^) control mice (Experiment 1). Secondly, we looked to confirm any AKU-related metabolite differences by assessing whether the direction of alteration was reversed while on the HGA-lowering drug nitisinone in *Hgd*^−/−^ mice or patients (Experiment 2). Thirdly, a metabolic flux experiment was carried out (Experiment 3), in which mice were injected with stable isotope ^13^C-labelled HGA to ascertain whether metabolites increased in *Hgd*^−/−^ were directly derived from HGA.

**Figure 1:**
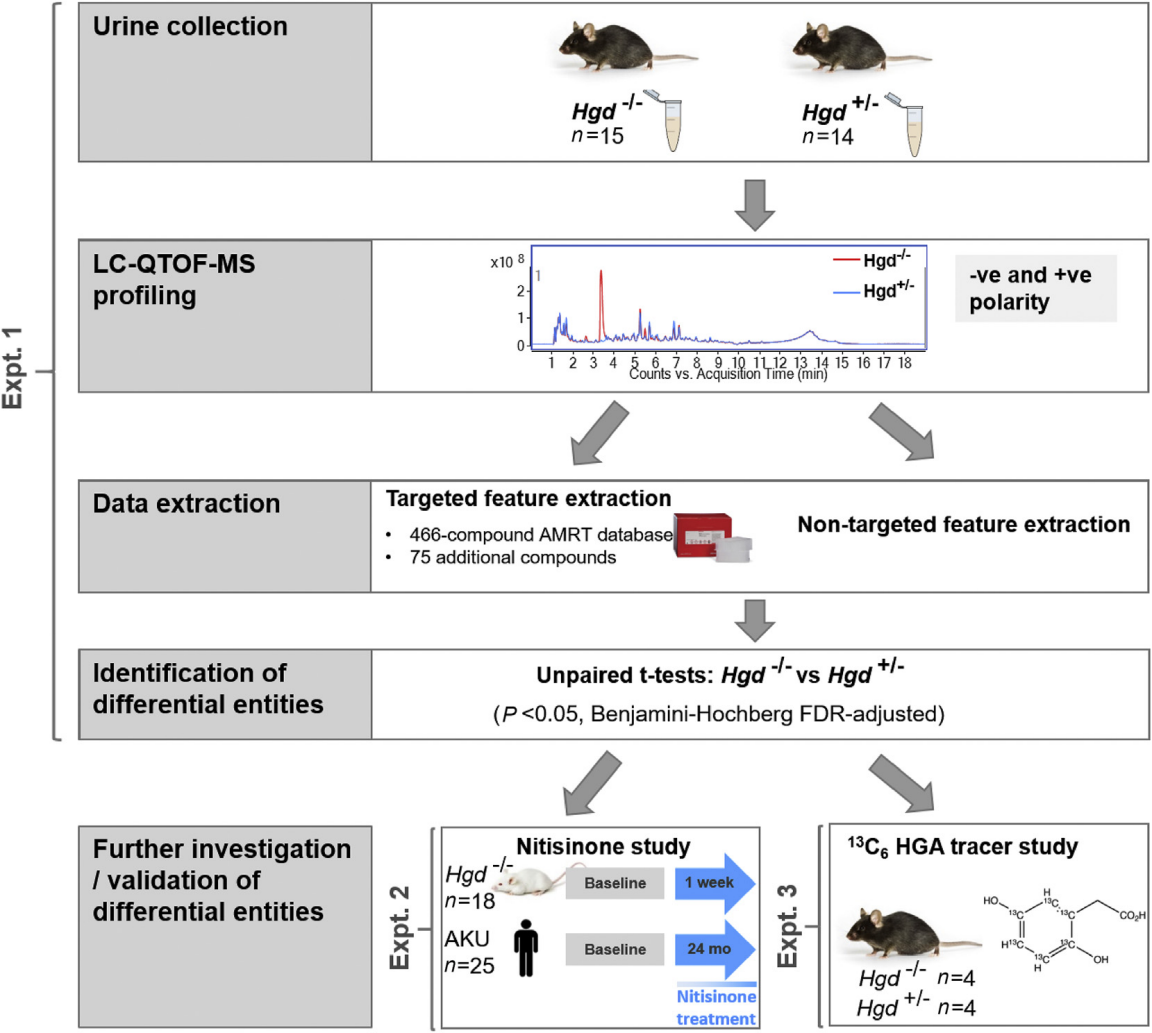
Schematic overview of the overall study design, incorporating Experiments 1–3. In Experiment 1, urine was collected from *Hgd*^−/−^ and *Hgd*^+/−^ mice and profiled by LC-QTOF-MS. Targeted and non-targeted feature extraction was performed on the data in parallel and subsequent unpaired *t*-tests were employed to identify differentially abundant compounds between *Hgd*^−/−^ and *Hgd*^*+/*−^. These compounds were then further investigated in LC-QTOF-MS data from two additional datasets; a previously published study examining the effect of nitisinone on the urine metabolome of *Hgd*^−/−^ BALB/c mice and patients with AKU^8^ (Experiment 2) and a plasma flux analysis using a ^13^C_6_ labelled HGA tracer (Experiment 3).

The overall aim of these experiments (Fig. 1) was to investigate in a controlled study the metabolic markers of disease in AKU and if metabolite alterations could elucidate possible novel disease mechanisms and thereby improve current strategies for monitoring the disease process and subsequent treatment response in patients with AKU.

## Materials and methods

### Animal housing and husbandry

All mice were housed in the University of Liverpool's Biomedical Services Unit under pathogen-free conditions, in cages of up to five mice, with 12-hr light/dark cycle, and food and water available *ad libitum*. Mice were drug/test-naïve at baseline in each experiment.

### Materials

Deionised water was purified in-house by DIRECT-Q 3UV water purification system (Millipore, Watford, UK). Methanol, acetonitrile, isopropanol (Sigma–Aldrich, Poole, UK), formic acid (Biosolve, Valkenswaard, Netherlands) and ammonium formate (Fisher Scientific, Schwerte, Germany) were LC/MS grade. ^13^C_6_ labelled HGA for metabolic flux analysis was purchased from Toronto Research Chemicals (Toronto, Canada).

### Urine collection and sample preparation for metabolomics (Experiment 1)

For metabolomic analysis of the targeted *Hgd* knockout phenotype,^10^ urine was collected from 15 *Hgd*^−/−^ (mean age ± SD 12.8 ± 0.1 weeks) and 14 *Hgd*^+/−^ (mean age 11.6 ± 0.3 weeks) male C57BL/6 mice. Drinking water was supplied *ad libitum* and the mouse urine was collected on a single-collection basis onto plastic wrap, pipetted into sample tubes and stored at −80 °C. Increased urinary HGA was expected to be the most marked metabolic alteration in *Hgd*^−/−^, but we were also interested to study the wider, potentially more subtle metabolic alteration both within the tyrosine pathway and in non-directly associated pathways. The number of mice studied was therefore considered appropriate to sufficiently power this experiment, given that mouse urine collection is a non-invasive procedure.

Pooled samples were created in each profiling experiment for quality control (QC) purposes. For *Hgd*^−/−^ and *Hgd*^+/−^ groups a separate representative pool was created by pooling 20 *μ*L of each individual urine sample. An overall pool was also created for each experiment by pooling equal proportions of the above group pools. Analysis of individual and pooled samples was performed following dilution of 1:9 urine:deionised water as previously described.^8^

### Investigating the effect of nitisinone on metabolites showing alteration in *Hgd*^*−/−*^ mice (Experiment 2)

The effect of nitisinone treatment on urinary metabolites altered in *Hgd*^−/−^ mice (Fig. 1, Experiment 1) was studied in the data from a previous profiling experiment described by Norman et al.^8^ These data were from urine from 18 BALB/c *Hgd*^−/−^ mice (mean age 27 ± 12 weeks, 9 female, 9 male) with *Hgd* disruption by ENU mutagenesis^11^ and from 25 patients attending the UK National Alkaptonuria Centre (NAC; mean age 51 ± 15 years, 13 female, 12 male). The disease phenotypes of *Hgd*^−/−^ mice from targeted knockout and ENU mutagenesis models are identical.^10^ Data were from urine collected at baseline, then on nitisinone at one week in mice (supplied *ad libitum* in drinking water at 4 mg/L) and at 24 months in patients (2 mg daily dose). These datasets were acquired under identical LC-QTOF-MS analytical conditions to those employed in the present study.

### Design of *in vivo* metabolic flux experiment and sample collection (Experiment 3)

Eight C57BL/6 mice were studied in the HGA metabolic flux experiment; four *Hgd*^−/−^ (mean age 56 ± 2.3 weeks, 1 female, 3 male) and four *Hgd*^+/−^ (mean age 58 ± 0 weeks, 4 female). A 1.96 mg/mL ^13^C_6_ HGA tracer solution was prepared in sterile saline and injected into the tail vein. Injection volume was adjusted for each mouse to achieve a final blood concentration of 1 mmol/L, assuming a total blood volume of 75 mL/kg.^12^ Venous tail bleed samples were then taken at 2, 5, 10, 20, 40 and 60 min post-injection, keeping sampling volumes within LASA guidelines.^12^ Mice were kept anaesthetised with isoflurane throughout the experiment. Blood was collected into Microvette 300 *μ*L lithium heparin capillary tubes (Sarstedt, Nümbrecht, Germany) and centrifuged at 1500×*g* for 10 min. Plasma supernatant was removed and stored at −80 °C prior to analysis. Individual plasma samples were analysed following 1:9 plasma:deionised water.

### LC-QTOF-MS analyses

Analysis of plasma and urine was performed on an Agilent 1290 Infinity HPLC coupled to an Agilent 6550 QTOF-MS equipped with a dual AJS electrospray ionization source (Agilent, Cheadle, UK). Reversed-phase LC was performed on an Atlantis dC18 column (3 × 100mm, 3 *μ*m, Waters, Manchester, UK) maintained at 60 °C. Mobile phase composition was (A) water and (B) methanol, both with 5 mmol/L ammonium formate and 0.1% formic acid. The elution gradient began at 5% B 0–1 min and increased linearly to 100% B by 12 min, held at 100% B until 14 min, then at 5% B for a further 5 min. MS data acquisition was performed in positive and negative ionisation polarity with mass range 50–1700 in 2 GHz mode with acquisition rate at 3 spectra/second. Sample injection volume was 2 *μ*L, and the autosampler compartment was maintained at 4 °C. Additional data acquisition parameters are detailed in Appendix 1.

Data-dependent tandem mass spectrometry (MS/MS) was performed on pooled urine samples, with compound hits from Experiment 1 as [M+H]^+^ and [M−H]^-^ accurate mass precursor ion targets; no more than six compound targets per injection. Fixed collision energies of 10, 20 and 40 V were applied. Acquisition rates were 6 spectra/second in MS and 4 spectra/second in MS/MS.

### Design of LC-QTOF-MS profiling analyses

Samples from Experiments 1 (*Hgd*^−/−^ *versus Hgd*^+/−^) and 3 (^13^C_6_ HGA metabolic flux analysis) were analysed in separate batches, each comprising negative then positive polarity. The analytical sequence of each profiling batch was designed according to published guidance^13^ and following the procedure described previously by Norman et al.^8^

### Data processing and statistical analyses

Mining of metabolite features in raw data was performed using two parallel approaches (Fig. 1, Experiment 1). A targeted approach was used to extract signals matching a 466-compound AMRT database previously generated in our laboratory from IROA Technology MS metabolite library of standards, accessible via https://doi.org/10.6084/m9.figshare.c.4378235.v2,^8^ or a compound database comprising accurate masses of additional compounds with potential relevance to AKU. A complementary non-targeted approach was used to extract unknown metabolites.

#### Targeted feature extraction

Targeted feature extraction was performed in Profinder (build 08.00, Agilent) using the chemical formulae of compounds from the AMRT database described above. Extraction parameters were accurate mass match window ±10 ppm and retention time (RT) window ±0.3 min. Allowed ion species were: H^+^, Na^+^, and NH_4_^+^ in positive polarity, and H^−^ and CHO_2_^-^ in negative polarity. Charge state range was 1–2, and dimers were allowed. ‘Find by formula’ filters were: score >60 in at least 60% of samples in at least one sample group (samples were grouped by *Hgd*^−/−^ or *Hgd*^+/−^ and pre- or on nitisinone).

Seventy-five additional metabolites of potential interest in AKU or from wider tyrosine metabolism were appended to the database for targeted extraction. Forty-three were from the following pathway databases available from Pathways to PCDL (build 07.00, Agilent): ‘citrate degradation’, ‘noradrenaline and adrenaline degradation’ and the ‘superpathway of phenylalanine, tyrosine and tryptophan biosynthesis’ (Appendix 2). Six metabolites were added as they were predicted to show potential alteration in *Hgd*^−/−^ due to association with tyrosine conjugation (acetyl-l-tyrosine, and γ-glutamyl-tyrosine) based on a previous publication^8^ or association with ochronotic pigment derived from HGA (2,5-dihydroxybenzaldehyde, benzoquinoneacetic acid, hipposudoric acid and norhipposudoric acid). Twenty-six metabolites were from a list of potential biotransformation products directly derived from HGA and compiled using the Biotransformation Mass Defects application (Agilent; Appendix 3). This tool provides a list of potential metabolic biotransformation products covering both phase I and II metabolism for a given compound based on empirical formula. The data were mined for these additional metabolites with putative identification by accurate mass (±5 ppm) only.

#### Non-targeted feature extraction

Non-targeted extraction was performed by recursive feature extraction in Profinder (build 08.00). Extraction parameters are detailed in Appendix 4.

#### Isotopologue feature extraction on data from ^13^C_6_ HGA metabolic flux analysis

Data from Experiment 3 (Fig. 1) were mined using batch isotopologue extraction in Profinder (build 08.00). Here, compounds that showed significant differences between *Hgd*^−/−^ and *Hgd*^+/−^ mice (Experiment 1) were investigated for potential association with the ^13^C_6_ HGA tracer by examining the relative abundances of their M+0 to M+6 isotopologues. Extraction was performed with accurate mass and RT match windows of ±5 ppm and ±0.3 min respectively against an AMRT database consisting only of these compound targets.

#### Data QC and statistical analyses

First, initial QC was performed on all datasets in Profinder by manual curation of the dataset to remove visually low quality peaks and to correct integration issues across the dataset where appropriate.

Urine profiling datasets were then exported from Profinder as .csv files and imported into Mass Profiler Professional (build 14.5, Agilent) for additional QC and subsequent statistical analyses. First, creatinine peak area from this analysis was used as an external scalar for each mouse sample to account for differences in urine concentration, as described previously.^8^ Additional QC was performed using data from pooled samples, which were interspersed throughout each analytical sequence. Compounds were retained if a) observed in 100% of replicate injections for at least one sample group pool, and b) peak area coefficient of variation (CV) remained <25% across replicate injections for each sample group pool. Statistically significant compounds were then identified in each dataset by *t*-tests; two sample *t*-tests for *Hgd*^−/−^ *versus Hgd*^+/−^ comparisons, and paired *t*-tests for pre- *versus* on nitisinone comparisons. Multiple testing correction was performed using Benjamini-Hochberg false discovery rate (FDR) adjustment. Fold changes (FC's) were log_2_-transformed and based on peak area. FC cut-off was not applied for compounds with FDR-adjusted *P* < 0.05, in order to retain compounds that showed consistent although relatively low magnitude abundance differences between comparison groups. Principal component analysis (PCA) was performed on each filtered dataset using four-component models.

#### *In vivo* metabolic flux data analysis

The results from isotopologue extraction on plasma ^13^C_6_ HGA metabolic flux data were reviewed visually in Profinder for clear evidence of an isotope label likely to be derived from HGA. This data review was performed for compound matches individually using the predicted number of ^13^C atoms derived from the HGA tracer based on chemical structure.

#### MS/MS data analysis

Raw MS/MS data files were processed in MassHunter Qualitative Analysis Workflows (Build 08.00), using the targeted MS/MS compound discovery algorithm. For compounds from the in-house AMRT database, MS/MS spectral library matching was performed against the METLIN metabolite PCDL accurate mass library (build 07.00), which contains over 30,000 compound entries, of which 1804 contain experimental MS/MS spectral data. For the HGA-derived compounds, for which no known chemical standards or compound library entries currently exist, structure identifications were performed using Agilent Molecular Structure Correlator (version B.07.00, build 31). This approach involves matching between accurate mass MS/MS fragment ions obtained from collision-induced dissociation MS with one or more proposed molecular structures based on a systematic bond disconnection approach.^14^

### Study approval

All animal work was carried out in accordance with UK Home Office Guidelines under the Animals (Scientific Procedures) Act, 1986 and with institutional approval.

Metabolomic analyses on patient samples was approved by the Royal Liverpool and Broadgreen University Hospital Trust's Audit Committee (audit no. ACO3836) and was part of the diagnostic service to patients attending the UK National Alkaptonuria Centre in Liverpool.

## Results

**Figure 2:**
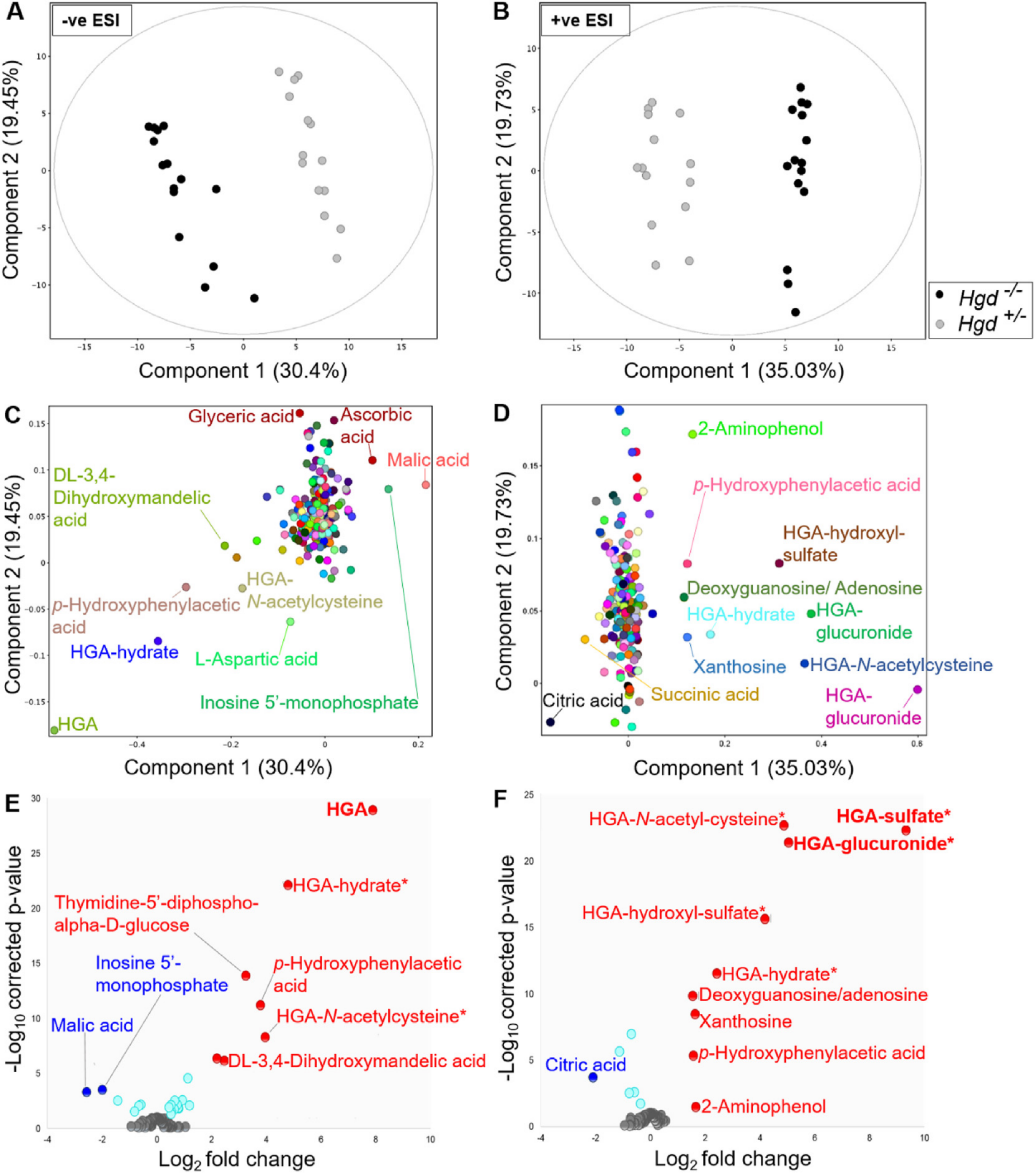
Clear differences between the urine metabolomes of *Hgd*^−/−^ and *Hgd*^+/−^ mice. (A–D) PCA on data from targeted feature extraction, with PCA plots showing separation between *Hgd*^−/−^ and *Hgd*^+/−^ mice by component 1 in A, negative, and B, positive ionisation polarities. Lower plots show the corresponding PCA loadings of metabolites on components 1 and 2 in C, negative, and D, positive polarity. (E, F) Volcano plots illustrating selection of statistically significant urinary metabolites between *Hgd*^−/−^ and *Hgd*^+/−^ mice based on *p*-value and fold change. (E) negative polarity; (F) positive polarity. Compounds with *P* < 0.05 (Benjamini-Hochberg FDR adjusted) and log_2_ fold change >1.5 are labelled, with red and blue indicating increased and decreased abundance, respectively, in *Hgd*^−/−^. Turquoise indicates adjusted *P* < 0.05 but log_2_ fold change <1.5. Bold text indicates that the increase observed in *Hgd*^−/−^ was confirmed in mouse plasma following injection with ^13^C_6_ HGA tracer. ∗ Compound not previously reported in the literature.

PCA showed clear separation in principal component 1 between the urine profiles of *Hgd*^−/−^ and *Hgd*^+/−^ mice from targeted (Fig. 2A, B) and non-targeted feature extraction. Results from targeted and non-targeted extraction are presented separately in the following sections (number of compounds obtained in each extraction method summarised in Appendix 5). Data from all animals in each experiment were included in the analysis.

### Targeted feature extraction

Targeted feature extraction was performed to search for metabolites based on AMRT (accurate mass ±10 ppm, RT ±0.3 min) or accurate mass (±5 ppm) alone. 27/250 and 15/243 metabolites showed abundance differences (FDR-adjusted *P* < 0.05) between *Hgd*^−/−^ and *Hgd*^+/−^ in negative and positive polarity, respectively. Table 1 shows the altered metabolites ranked by FC. PCA loadings plots (Fig. 2C, D) and volcano plots (Fig. 2E, F) show that the greatest differences between *Hgd*^−/−^ and *Hgd*^+/−^ urine were in metabolites associated with HGA in negative and positive polarity. HGA and six predicted HGA biotransformation products were markedly elevated in *Hgd*^−/−^. Interestingly, HGA-sulfate (FC = 9.3, *P* < 0.0001) showed a greater FC increase than HGA (FC = 7.9, *P* < 0.0001). Other HGA products increased with FC > 1.5 and *P* < 0.0001 were HGA-glucuronide, HGA-hydroxylsulfate, HGA-*N*-acetylcysteine and HGA-hydrate. Acetyl-HGA was elevated in *Hgd*^−/−^ (FC = 2.3, *P* < 0.0001), but did not pass QC filtering by CV <25% across replicate injections of pooled QC samples; a decrease in signal across the run indicated a stability issue (despite the auto-sampler being maintained at 4 °C).

**Table 1:**
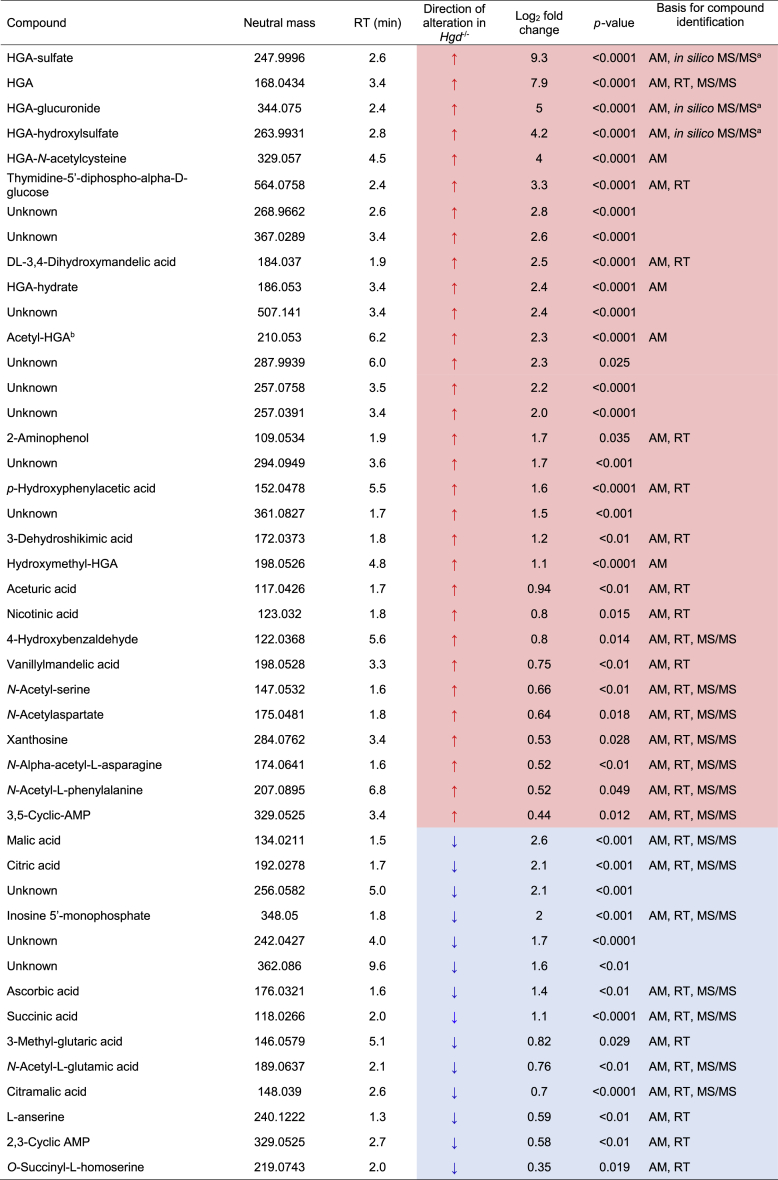
Summary of urinary metabolites showing altered abundance in *Hgd*^−/−^ mice.

Direction of alteration and log_2_ fold change is indicated in *Hgd*^−/**−**^ (relative to *Hgd*^+/**−**^); red and blue shading indicates increased and decreased abundance in *Hgd*^−/**−**^, respectively. *p*-values are false discovery rate adjusted. Where compounds were significantly different in positive and negative polarity, the result with the lowest fold change is provided. For compounds identified by accurate mass (AM) and retention time (RT), match criteria were accurate mass (±10 ppm) and RT (±0.3mins) against a database generated in-house from metabolite standards. Compound identifications based on accurate mass match alone were with a mass window ±5 ppm.

^a^MS/MS compound identification based on matching experimental spectra with *in silico* fragmentation data, using Molecular Structure Correlator (MSC) with score threshold >65%; all other MS/MS matches were against spectra from the MassHunter METLIN metabolite PCDL accurate mass library (build 07.00). ^b^Acetyl-HGA failed quality control filtering (CV >25% across replicate injections of QC pooled samples; due to suspected compound stability issue over analysis period).

Excluding HGA, 26 significantly altered compounds were AMRT-matched with metabolites from the 466-compound library developed in-house. The most significantly altered (*P* < 0.05 and log_2_ FC > 1.5) of these were: thymidine-5′-diphospho-alpha-d-glucose, DL-3,4-dihydroxymandelic acid, 2-aminophenol, *p*-hydroxyphenylacetic acid (increased in *Hgd*^−/−^), malic acid, citric acid and inosine 5′-monophosphate (decreased in *Hgd*^−/−^).

MS/MS fragmentation data were acquired to confirm the compound identifications of the significantly altered metabolites. For the HGA-derived metabolites, the proposed structures (Fig. 4) were imported into the in-house AMRT compound library as .mol files for matching of experimental MS/MS spectra against *in silico* fragmentation predictions using Molecular Structure Correlator (MSC). MSC match scores >85% were obtained for HGA-glucuronide (91.2%), HGA-sulfate (86.7%) and HGA-hydroxylsulfate (85.9%), strongly supporting the chemical structures proposed for these compounds. Intermediate scores were obtained for the proposed structure for acetyl-HGA (54.3%) and hydroxymethyl-HGA (53.9%). Lower MSC scores were obtained for the structures proposed for HGA-*N*-acetylcysteine (32.4%) and HGA-hydrate (20.3%). The MSC score obtained for each of these HGA-derived structures was either the only compound structure match or was greater than the highest scoring match from the MassHunter METLIN metabolite library. For the significantly altered metabolites that were not HGA biotransformation products, 15 of the AMRT-matched compounds were confirmed by MS/MS spectral library match, with a match threshold of ≥65% (Table 1 & Appendix 6).

**Figure 3:**
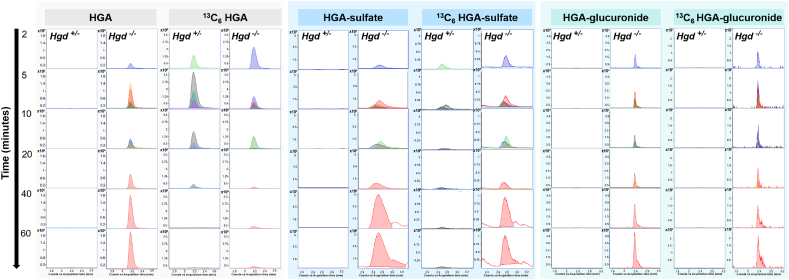
Isotopologue extraction results on plasma from the *in vivo* metabolic flux experiment using injected ^13^C_6_-labelled homogentisic acid (HGA). Data shown are from *Hgd*^−/−^ and *Hgd*^+/−^ samples taken at intervals of 2, 5, 10, 20, 40 and 60 (when possible) min after injection. Extracted ion chromatograms (EIC’s) represent the M+0 (native compound) and M+6 (^13^C_6_-labelled form) isotopologue signals for HGA, HGA-sulfate and HGA-glucuronide. EIC’s show clear M+6 peaks for these compounds following injection (but only from *Hgd*^−/−^ mice for HGA-glucuronide), confirming that they are derived from the labelled HGA.

**Figure 4:**
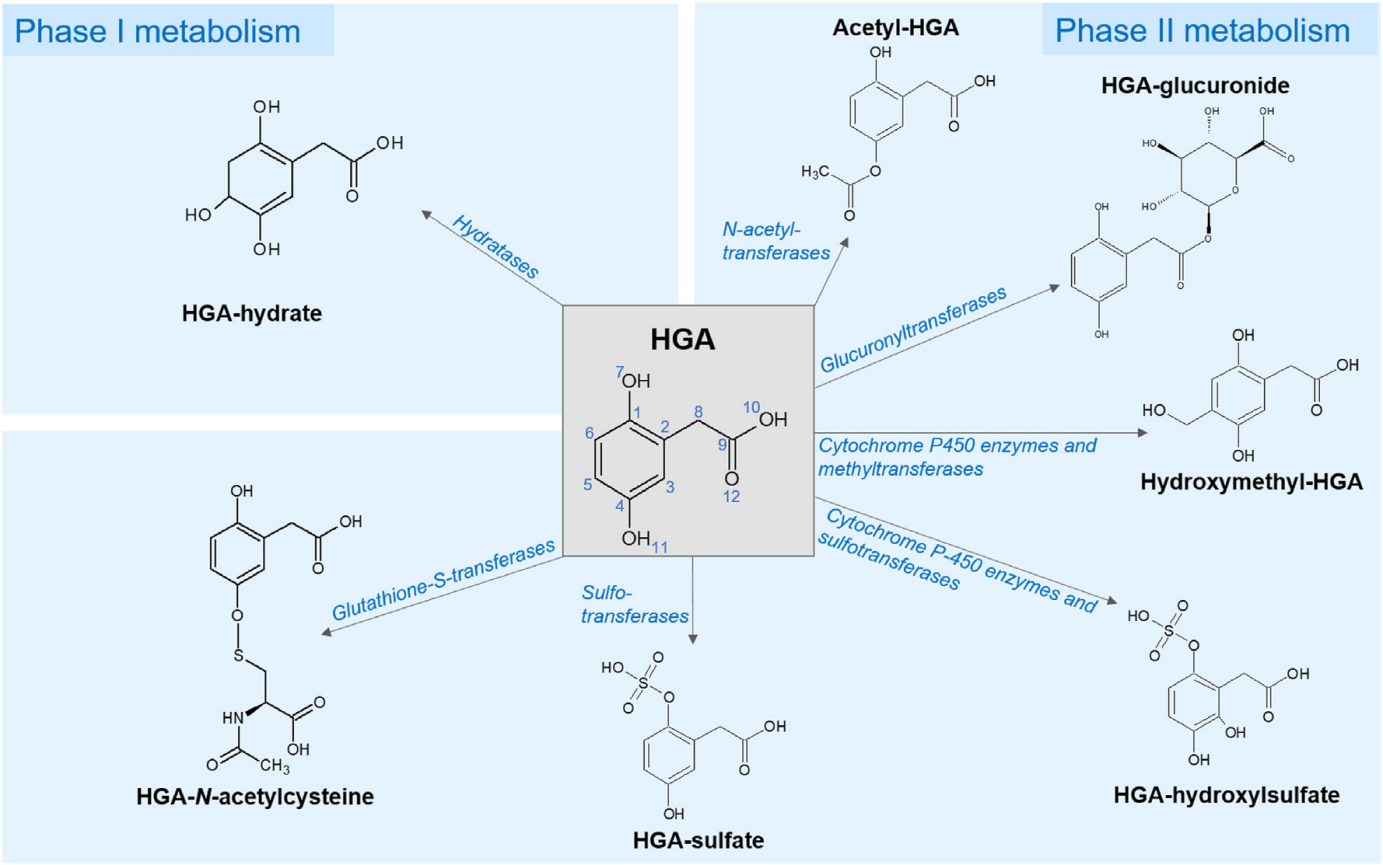
Predicted structures of newly-identified HGA biotransformation products resulting from phase I and II metabolism. Predicted structures were the closest matches against the acquired experimental MS/MS data, based on scores obtained using Agilent Molecular Structure Correlator (MSC). The proposed sites for metabolism/conjugation were based on match scores obtained for a list of possible candidates using a combination of MSC and CFM-ID 3.0^53^*in silico* fragmentation modelling tools.

### Non-targeted feature extraction

Non-targeted feature extraction yielded 359 and 213 compounds post-QC in negative and positive polarity respectively; mass range = 54–3108 Da and RT range = 1.1–11.5 min. Comparison of *Hgd*^−/−^ and *Hgd*^+/−^ revealed compounds with clear abundance differences (*P* < 0.05, log_2_ FC > 1.5); 9 in negative polarity (6 increased in *Hgd*^−/−^, 3 decreased in *Hgd*^−/−^) and 2 in positive polarity (both increased in *Hgd*^−/−^). The mass range of these significant compounds was 242–507 Da, and RT range was 1.7–9.6 min (Table 1). Searching of the MassHunter METLIN metabolite PCDL accurate mass library (build 07.00) yielded no matches for these compounds based on library MS/MS spectral matching or accurate mass (<5 ppm) alone.

### The effect of nitisinone treatment on metabolites altered in *Hgd^−/−^* mice

To investigate the effect of nitisinone treatment on the metabolites shown to be altered here in *Hgd*^−/−^, they were searched in the data from previous mouse and human urine profiling experiments (Experiment 2; Fig. 1).^8^

Eight compounds from targeted feature extraction that were altered in *Hgd*^−/−^ *versus Hgd*^+/−^ mice were significantly altered in the opposite direction in urine from both *Hgd*^−/−^ mice and patients with AKU on nitisinone (based on peak area pre- *versus* on nitisinone, Benjamini-Hochberg FDR-adjusted *P* < 0.05; Table 2); seven decreased, and one increased. The seven decreased metabolites were HGA, the HGA biotransformation products HGA-sulfate, HGA-glucuronide, HGA-hydrate and hydroxymethyl-HGA, and also xanthosine and 3,5-cyclic-AMP. The increased metabolite was 3-methyl-glutaric acid. Interestingly, *p*-hydroxyphenylacetic acid and 4-hydroxybenzaldehyde were increased both in *Hgd*^−/−^ *versus Hgd*^+/−^ and also on nitisinone in *Hgd*^−/−^ mice and patients.

**Table 2:**
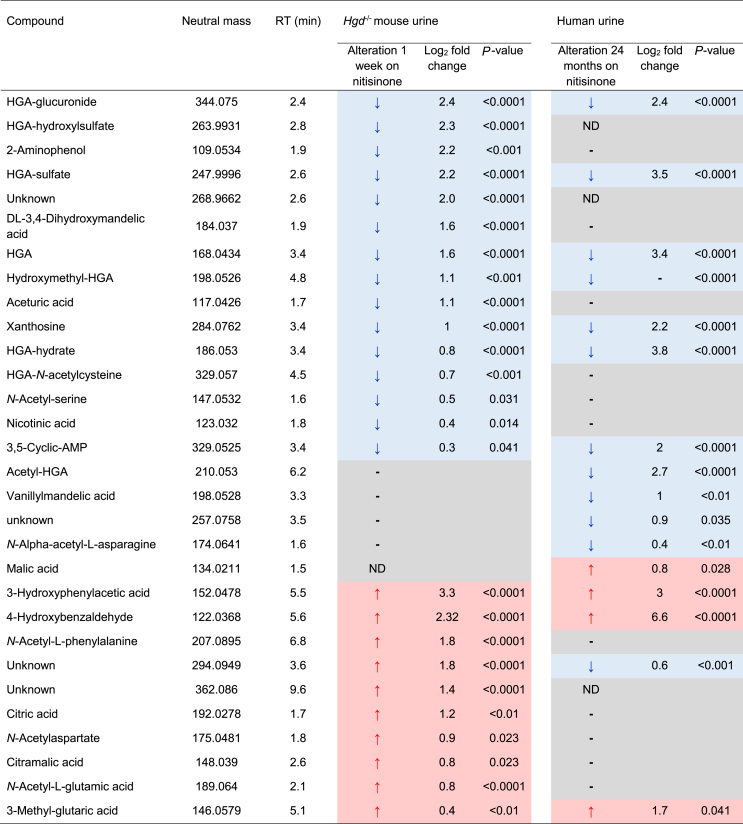
The effect of nitisinone treatment in *Hgd*^−/−^ mice and patients with AKU on the abundance of urinary metabolites altered in *Hgd*^−/−^*vs.Hgd*^+/−^ mice.

The compounds that showed differences between *Hgd*^−/**−**^ vs *Hgd*^+/**−**^ mice (Table 1) were examined in two additional datasets. Paired t-tests were employed to compare the abundances at baseline *versus* 1 week on nitisinone (4 mg/L, in drinking water) for *Hgd*^−/**−**^ mice and 24 months on 2 mg daily nitisinone for patients with AKU. Only compounds with false discovery rate adjusted *P* < 0.05 pre-vs on nitisinone in mouse or human are displayed. Direction of alteration and log_2_ fold change are indicated; red and blue shading indicates increased and decreased abundance on nitisinone, respectively. Where compounds were significantly different in positive and negative polarity, the result with the lowest fold change is provided. Note: no fold change indicated for hydroxymethyl-HGA in humans, as this compound was not detected for any patient on nitisinone.

ND: compound not detected at baseline or on nitisinone.

Nineteen compounds that were altered in *Hgd*^−/−^ *versus Hgd*^+/−^ mice were significantly altered in the opposite direction on nitisinone in urine from either *Hgd*^−/−^ mice (*n* = 14; 9 decreased, 5 increased on nitisinone) or patients (*n* = 5; 4 decreased, 1 increased on nitisinone) only (Table 2). These compounds comprised the remaining HGA biotransformation products, which were all decreased on nitisinone. On nitisinone, acetyl-HGA was decreased in patients only, and HGA-hydroxyl-sulfate and HGA-*N*-acetylcysteine were decreased in mice only.

### Confirmation of HGA biotransformation products by ^13^C_6_ HGA metabolic flux analysis (Experiment 3)

Data from isotopologue extraction were compared between plasma collected from the same mice across the time intervals available (2–60 min). The M+6 isotopologue was of particular interest as mice were injected with ^13^C_6_-labelled HGA. A clear M+6 peak for HGA was observed over the sampling time course in plasma from *Hgd*^−/−^ and *Hgd*^+/−^ mice, although the signal decreased from 10 to 20 min post-injection (Fig. 3). As indicated in Figure 2 (compounds in bold text), two HGA biotransformation products that were increased in *Hgd*^−/−^ urine were observed with clear M+6 peaks over the time course; HGA-glucuronide and HGA-sulfate. These data confirm that the compounds are derived from HGA. HGA-sulfate showed a similar time course profile to HGA; the native M+0 isotopologues were absent from *Hgd*^+/−^ plasma at all time points, in contrast to the M+6 isotopologue whose profile appeared to closely follow that of the HGA M+6 peak over the time course in both *Hgd*^−/−^ and *Hgd*^+/−^ (Fig. 3). For HGA-glucuronide, the native M+0 isotopologue was also absent from *Hgd*^+/−^ plasma, but the M+6 isotopologue was only observed in *Hgd*^−/−^ (Fig. 3), indicating that glucuronidation of the HGA tracer was evident only for *Hgd*^−/−^ mice.

## Discussion

The data reported form part of the first recorded metabolome-wide comparison obtained from untreated AKU *versus* non-AKU animal models and shows, for the first time, that the overproduced HGA in AKU undergoes predominantly phase II metabolic biotransformations. Phase II metabolism involves conjugation reactions to form sulfate, glucuronide, glutathione, mercapturic acid, amino acid, methyl and acetyl conjugates.^15^^,^^16^ The predominant sulfation and glucuronidation biotransformations observed for HGA are also essential aspects of metabolism of other phenolic acids, bile acids, steroids and numerous other endogenous biochemicals. The phase I metabolism reactions of hydroxylation, oxidation, reduction and hydrolysis were relatively minor. Further, our data indicate that the biochemical consequences of HGD deficiency extend beyond tyrosine metabolism.

### HGA biotransformation products from phases I and II metabolism

The clearest differences between the urine metabolomes of *Hgd*^−/−^ *versus Hgd*^+/−^ mice were in HGA and seven previously unreported HGA-derived biotransformation products, increased in *Hgd*^−/−^*.* The decreased output in HGA-sulfate, HGA-glucuronide, HGA-hydrate, acetyl-HGA and hydroxymethyl-HGA for patients on treatment with nitisinone (Experiment 2) indicates that these HGA phase II biotransformations occur in human AKU. The detection of ^13^C_6_-labelled forms of HGA-glucuronide and HGA-sulfate in plasma following ^13^C_6_-HGA injection in mice (Experiment 3) confirm products derived from HGA. The ^13^C_6_-HGA-sulfate was observed both in *Hgd*^−/−^ and *Hgd*^+/−^ mice, following the profile of the ^13^C_6_-HGA across the sampling time course. The ^13^C_6_-HGA-glucuronide was observed only in *Hgd*^−/−^, suggesting prior upregulation of glucuronyltransferase activity was required to enhance HGA clearance in AKU.

**Figure 5:**
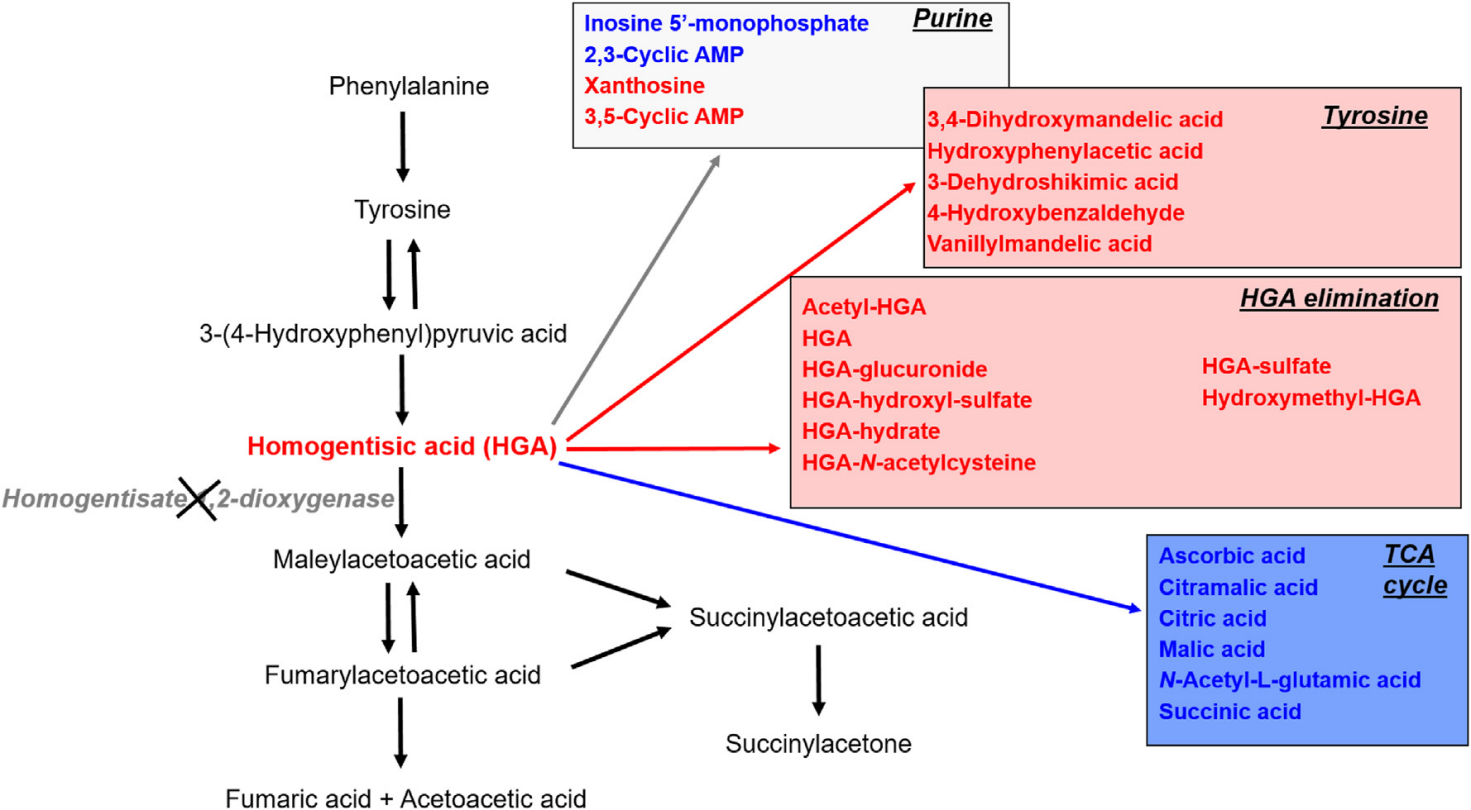
Summary of metabolites altered in *Hgd*^−/−^ mouse urine grouped by their associated pathways. Left: the tyrosine degradation pathway showing lack of the enzyme HGD in AKU and the consequential increase in HGA. Right (boxes): observed metabolite alterations grouped by pathway; red and blue indicate increased and decreased abundance respectively. Tyrosine metabolites, including HGA and HGA biotransformation products, were elevated. Metabolites associated with the TCA cycle were decreased. A combination of increased and decreased abundance was observed for purine pathway metabolites.

The putative structures of these newly identified biotransformation compounds, together with the enzyme classes for their formation are shown in Figure 4, although the exact enzymes are not yet described. The proposed biotransformation compound structures are the closest known matches, based on correlation between the observed experimental MS/MS spectra and the accurate mass fragment ions predicted *in silico* from the 30,000+ metabolite structures in the Metlin and in-house compound libraries. In the absence of reference standards for these compounds, the MS/MS data confirm the structures proposed for HGA-glucuronide, HGA-sulfate and HGA hydroxylsulfate, with MSC scores >85%. To our knowledge, these are the first data on HGA metabolism not involving conversion to maleylacetoacetic acid in the traditional tyrosine degradation pathway (Fig. 5).

The markedly increased output of the HGA-glucuronide (FC = 5) and HGA-sulfate (FC = 9.3), as well as HGA (FC = 7.9), in AKU *Hgd*^−/−^ mice has implications for the regulation of plasma HGA and products. Despite efficient renal excretion of HGA-related compounds in AKU,^17^ the circulating HGA remains elevated; mean serum HGA concentration is 30 *μ*mol/L in untreated AKU^18^ and <1.5 *μ*mol/L in healthy subjects.^19^^,^^20^ The increased plasma HGA persists as the primary toxic agent in AKU; its oxidation produces ochronotic pigment, which becomes bound within the extracellular matrix of collagenous tissues. This process of damage is thought to occur via HGA oxidation to highly reactive benzoquinone and free radical intermediates.^5^ These species are capable of inducing oxidative changes to proteins and lipids,^21^^,^^22^ are known to self-perpetuate osteoarthropathy associated with ochronosis^5^ and account for cases of acute fatal metabolic consequences reported in the literature.^23^ The data reported here show that metabolism of HGA by phase II biotransformations, particularly glucuronidation and sulfation reactions, is a major route in an attempt to render the increased HGA chemically inert (detoxify), in effect to protect from consequences of increased benzoquinone production. The HGA-derived products observed must be included in metabolic profiling in AKU patients to understand the total production of HGA and how this impacts on predicting the amount of HGA-derived pigment produced *in vivo*.

It is not clear whether the HGA biotransformations described are simply unmasked in AKU due to the markedly increased HGA, or whether they are actively and exclusively recruited in AKU for HGA detoxification. Nevertheless, identification of the specific enzymes that catalyse the biotransformations could inform future AKU therapeutic interventions aimed at enhancing HGA metabolism. Glucuronidation, for example, is quantitatively one of the most important phase II biotransformation reactions, performed by 15 UPD-glucuronosyltransferase enzymes for conjugation of a large number of exogenous and endogenous compounds in humans.^24^^,^^25^ A number of agents, including naturally occurring dietary compounds, are known to be potent inducers of UPD-glucuronosyltransferases and other phase II enzymes.^26^, ^27^, ^28^ There are encouraging examples of targeting specific enzymes of phase II metabolism in other conditions of endogenous compound accumulation, including UDP-glucuronosyltransferase 1A1 in neonatal hyperbilirubinaemia^29^ and sulfotransferases in oestrogen-dependent breast cancer.^30^

The liver is the primary site of phase I and II metabolism, both by tissue mass and activity.^31^ Extrahepatic metabolism in tissues such as the kidney and intestinal epithelium is also significant.^32^ It is possible that site-specific enzyme activity explains the interesting finding that conjugation products from the ^13^C_6_-labelled HGA were observed mainly for glucuronidation and sulfation. The other biotransformation products observed in urine were not detected in the plasma tracer experiment, despite their clear concomitant urinary reduction on nitisinone confirming direct association with HGA. In the metabolic flux experiment, HGA was administered intravenously, which is likely to favour formation of products from hepatic metabolism. It would be interesting therefore to give HGA by various routes of delivery and trace its metabolism in various tissues and biofluids to assess the metabolic activity contributed by different anatomical compartments. It is also worth noting that the glycine conjugate of HGA, a potential HGA biotransformation product, was not detected in *Hgd*^−/−^; glycine conjugation is a known detoxification mechanism of a number of other aromatic acids.^33^^,^^34^

The observation of marked elevations in five of the seven HGA-derived compounds which were initially discovered in mice, including the major biotransformation products from HGA sulfation and glucuronidation, in untreated patients with AKU (Experiment 2) supports use of the *Hgd*^−/−^ mouse as an accurate model of human AKU biochemistry. Our preclinical studies of the *Hgd*^−/−^ mouse have helped us to understand the metabolic and wider pathophysiological features of AKU in humans, the natural course of the disease and its response to treatment. The mice studied in Experiment 1 were male only in order to enable a highly controlled and powered examination of the metabolic impact of *Hgd* knockout. The inclusion of the previously published data from equal male and female *Hgd*^−/−^ mice in Experiment 2 confirms that the metabolic alterations observed for AKU mice in Experiment 1 are not restricted to males. Further, in Experiment 3 we observed both native non-labelled and ^13^C-labelled HGA-glucuronide and HGA-sulfate products for male and female *Hgd*^*−/−*^ mice. As noted in our published discussion of the dataset featured in Experiment 2, the metabolomic difference between urine from male and female mice is largely attributable to the markedly increased urinary histamine in female mice, and no difference in metabolism of tyrosine or HGA.^8^

### Associated alteration to tyrosine, purine and TCA cycle metabolism in AKU

This study showed for the first time that in untreated AKU there is alteration to tyrosine, purine and TCA cycle metabolites (Fig. 5). Previous metabolomic studies in AKU have focused solely on the impact of nitisinone on the metabolome.^8^^,^^9^^,^^35^, ^36^, ^37^ Nitisinone reversibly inhibits hydroxyphenylpyruvic acid dioxygenase (HPPD; E.C. 1.13.11.27), the enzyme that produces HGA, and it is currently the most effective treatment for AKU. Nitisinone reduces plasma and urine HGA concentrations,^18^^,^^19^^,^^38^, ^39^, ^40^ completely arrests ochronosis in AKU mice^11^^,^^41^ and more recently was shown to decrease ochronosis and improve clinical signs of AKU in patients enrolled in SONIA 2, the 4-year phase 3 international randomised controlled trial of nitisinone in AKU (ClinicalTrials.gov identifier: NCT01916382).^42^ We showed previously in serum and urine that nitisinone induced an extended network of metabolic alteration within tyrosine and neighbouring pathways, including tryptophan, purine and TCA cycle.^8^^,^^9^ This alteration is a concern in AKU, and particularly in hereditary tyrosinaemia type-1, another inherited disorder of tyrosine metabolism, in which nitisinone treatment is essential from early infancy.^43^

The increases in tyrosine metabolites reported here, excluding HGA, were unexpected in untreated AKU. Increases in metabolites upstream of HPPD previously reported in nitisinone-treated AKU were thought to be a direct consequence of the inhibition of HPPD by nitisinone and the consequential hypertyrosinaemia.^8^^,^^9^ For the first time, the present data show that targeted *Hgd* disruption in *Hgd*^−/−^ mice induces metabolic changes upstream of HGA, despite no increase in tyrosine, hydroxyphenylpyruvic acid or hydroxyphenyllactic acid. The cause of these unexpected changes to tyrosine and peripheral neurotransmitter metabolism in untreated AKU is not clear. At the supraphysiological concentrations observed in AKU, HGA could potentially act on other enzymes of neurotransmitter metabolites and alter their activity. Alternatively, the changes could relate to an unknown feature of the disease.

In nitisinone-treated AKU, the purine metabolite changes previously reported were mainly decreased concentrations.^8^ The present data indicate a more complex pattern of purine alteration in untreated AKU; increased 3,5-cyclic AMP and xanthosine, and decreased 2,3-cyclic AMP and inosine 5′-monophosphate. Purine catabolism is important in the homeostatic response to various states of mitochondrial oxidative stress, with shifts occurring to favour breakdown to xanthine and uric acid, the final breakdown products of purines.^44^^,^^45^ The increased xanthosine and decreases in the upstream purine pathway metabolites 2,3-cyclic AMP and inosine 5′-monophosphate reported here for *Hgd*^−/−^ mice is consistent with a shift in purine catabolism as a protection mechanism against HGA-induced oxidative stress in untreated AKU, as xanthine derivatives have anti-oxidant properties.^46^ There is growing evidence that HGA-induced oxidative stress is an important feature of AKU (see Ref. ^22^ for an extensive review on this subject), and that it exacerbates ochronosis, the central AKU disease process.^47^ The increased xanthosine observed here is likely a new metabolic marker of HGA-induced oxidative stress in AKU; urinary xanthosine is markedly increased in other oxidative diseases, including gout,^48^ chronic kidney disease and diabetes nephropathy,^49^ and it is markedly decreased following administration of nephroprotective therapy.

Decreases in TCA-related metabolites in *Hgd*^−/−^ is the first indication of perturbed energy metabolism in untreated AKU. Decreases in citramalic acid, citric acid and *N*-acetyl-l-glutamic acid were reversed on nitisinone in *Hgd*^−/−^, suggesting for the first time that nitisinone is at least partially restorative. Blood and urine concentrations of TCA metabolites are generally considered to directly reflect overall TCA cycle activity.^50^ Inhibited TCA cycle activity could be due to overall mitochondrial biogenesis, decreased expression of genes encoding TCA cycle enzymes, dysregulation of enzyme activity or reduced substrate availability. The latter explanation seems the most likely in AKU, in which HGD deficiency prevents further metabolism of HGA to the TCA cycle intermediate fumaric acid. Decreased bioavailability of fumaric acid may then explain the decreases observed for other TCA cycle metabolites. Previous analyses have investigated potential changes to TCA cycle metabolites in AKU, but these were limited to studying the effect of nitisinone; serum concentrations of TCA metabolites succinic acid and *α*-ketoglutaric acid were decreased in patients with AKU on nitisinone.^9^ The significance of these decreases is not known, and further studies should investigate whether the alteration to urinary TCA cycle metabolites reported here also applies to serum in untreated AKU.

Decreased urinary citrate is a well-known risk factor for kidney stone formation. It is possible that the decreased citric acid reported here for *Hgd*^−/−^ mice is associated with the increased risk of kidney stones in AKU.^51^ Hypocitraturia is generally defined as citric acid excretion <320 mg (1.67 mmol) per day in adults.^52^ The present profiling data are semi-quantitative; targeted quantitative assays are required to determine exact urinary citric acid concentrations in AKU patients and the additional therapeutic value of nitisinone in this regard.

## Conclusions

In conclusion, we have shown that targeted homozygous disruption to the *Hgd* gene induces previously unknown metabolite changes in a complex network of pathways associated with altered tyrosine metabolism. These changes include the biotransformation of HGA by mainly phase II metabolic processes and relatively little by phase I. This observation will encourage further as yet unexplored potential treatment targets for detoxification of HGA, the culprit molecule in AKU. The wider take-home message of this study is that inherited metabolic diseases, such as AKU, present unique windows into metabolism that can uncover associations between metabolic pathways in physiology and biochemistry more generally.

## Author contributions

**BPN, ASD:** Conceptualization, Methodology, Formal analysis, Investigation, Data curation, Writing – Original draft preparation, Writing – Review and Editing, Visualization. **JHH, HS, PJW, NGB, ATH, AMM:** Investigation, Writing – Review and Editing. **JCJ:** Resources, Writing – Review & Editing. **GBG:** Resources, Writing – Review and Editing, Supervision. **NBR, LRR, JAG:** Supervision, Writing – Review and Editing.

## Conflict of interests

The authors declared no conflict of interests.

## Funding

BPN is funded by the 10.13039/501100000836University of Liverpool, Royal Liverpool University Hospitals Trust and Agilent Technologies UK Ltd. ASD is funded through a 10.13039/501100000272National Institute for Health Research Doctoral Research Fellowship (No. HCS DRF-2014-05-009). JHH is funded by the Alkaptonuria Society. This article presents independent research partially funded by the 10.13039/501100000272NIHR. The views expressed are those of the author(s) and not necessarily those of the NHS, the NIHR or the Department of Health and Social Care.

## Data Availability

The datasets generated during and/or analysed during the current study are available in the MetaboLights repository. Study identifier: MTBLS2525 [https://www.ebi.ac.uk/metabolights/MTBLS2525].
