# Supplementary material for: Metabolomic studies in the inborn error of metabolism alkaptonuria reveal new biotransformations in tyrosine metabolism
Source: Genes Dis. 2021 Feb 22;9(4):1129–42. doi: 10.1016/j.gendis.2021.02.007 (PMC9170613; doi:10.1016/j.gendis.2021.02.007)
Supplement: Multimedia component 3 [file mmc3.docx]

**Appendix 3.** **Additional biotransformation product compound targets for feature extraction (2).** Non-AMRT database metabolite products from predicted phase I and II biotransformations of homogentisic acid (HGA).

| Biotransformation | Formula | Mass | Phase |
| --- | --- | --- | --- |
| HGA Alcohols Dehydration | C8H6O3 | 150.03169 | I |
| HGA Alkene to Epoxide | C8H8O5 | 184.03717 | I |
| HGA Decarboxylation | C7H8O2 | 124.05243 | I |
| HGA Demethylation | C7H6O4 | 154.02661 | I |
| HGA Demethylation and Hydroxylation | C7H6O5 | 170.02152 | I |
| HGA Demethylation and Methylene to Ketone | C7H4O5 | 168.00587 | I |
| HGA Demethylation and two Hydroxylations | C7H6O6 | 186.01644 | I |
| HGA Hydration, Hydrolysis (Internal) | C8H10O5 | 186.05282 | I |
| HGA Hydroxylation and Desaturation | C8H6O5 | 182.02152 | I |
| HGA Hydroxymethylene Loss | C7H6O3 | 138.03169 | I |
| HGA (*O*, *N*, *S*) Methylation | C9H10O4 | 182.05791 | II |
| HGA 2x Sulfate Conjugation | C8H8O10S2 | 327.95589 | II |
| HGA Acetylation | C10H10O5 | 210.05282 | II |
| HGA Cysteine Conjugation | C11H15NO6S | 289.06201 | II |
| HGA Cysteine Conjugation and Desaturation | C11H13NO6S | 287.04636 | II |
| HGA Cysteine Glycine Conjugation | C13H18N2O7S | 346.08347 | II |
| HGA Glucuronide Conjugation | C14H16O10 | 344.07435 | II |
| HGA Glutamine Conjugation | C13H16NO6 | 282.09776 | II |
| HGA Glycine Conjugation | C10H11NO5 | 225.06372 | II |
| HGA Hydroxylation + Glucuronide | C14H16O11 | 360.06926 | II |
| HGA Hydroxylation and Methylation | C9H10O5 | 198.05282 | II |
| HGA Hydroxylation and Sulfation | C8H8O8S | 263.99399 | II |
| HGA *N*-Acetylcysteine Conjugation | C13H17NO7S | 331.07257 | II |
| HGA *N*-Acetylcysteine Conjugation and Desaturation | C13H15NO7S | 329.05692 | II |
| HGA Sulfate Conjugation | C8H8O7S | 247.99907 | II |
| HGA Taurine Conjugation | C10H13NO6S | 275.04636 | II |
