# Supplementary material for: Metabolomic studies in the inborn error of metabolism alkaptonuria reveal new biotransformations in tyrosine metabolism
Source: Genes Dis. 2021 Feb 22;9(4):1129–42. doi: 10.1016/j.gendis.2021.02.007 (PMC9170613; doi:10.1016/j.gendis.2021.02.007)
Supplement: Multimedia component 6 [file mmc6.docx]

**Appendix 6. Summary of MS/MS compound identification scores.** MS/MS identifications were based on spectral matching against data from the MassHunter METLIN metabolite PCDL accurate mass library (build 07.00), accessed through Agilent PCDL Manager (build 08.00). For newly-identified HGA conjugate metabolites for which no known chemical standards or spectral data exist, compound identification was based on matching experimental MS/MS spectra against *in silico* predicted fragmentation patterns for the predicted structures, using Agilent Molecular Structure Correlator (version B.07.00, build 31).

| Compound | MS/MS library spectral match score (%) | | |  | Molecular Structure Correlator score  (%) |
| --- | --- | --- | --- | --- | --- |
|  | Forward^a^ | Reverse^a^ | Composite |  |  |
| HGA-sulfate | - | - | - |  | 86.7 |
| HGA | 91.9 | 97 | 97 |  | - |
| HGA-glucuronide | - | - | - |  | 91.2 |
| HGA-hydroxylsulfate | - | - | - |  | 85.9 |
| HGA-*N*-acetylcysteine | - | - | - |  | 32.4 |
| HGA-hydrate | - | - | - |  | 20.3 |
| Actyl-HGA | - | - | - |  | 54.3 |
| Hydroxymethyl-HGA | - | - | - |  | 53.9 |
| 4-Hydroxybenzaldehyde | 83.6 | 87.4 | 85.2 |  | - |
| N-Acetyl-serine | 82.2 | 91.5 | 92.04 |  | - |
| N-Acetylaspartate | 63.1 | 89.1 | 89.37 |  | - |
| Xanthosine | 83.5 | 96.6 | 96.8 |  | - |
| N-Alpha-acetyl-l-asparagine | 20.7 | 67.9 | 67.9 |  | - |
| N-Acetyl-L-phenylalanine | 45.4 | 96.1 | 96.1 |  | - |
| 3,5-Cyclic-AMP | 97.4 | 53.8 | 97.7 |  | - |
| Malic acid | 51.8 | 94.4 | 94.5 |  | - |
| Citric acid | 93.9 | 89.8 | 93.9 |  | - |
| Inosine 5’-monophosphate | 69.4 | 69.4 | 68.4 |  | - |
| Ascorbic acid | 84.5 | 94.1 | 94.25 |  | - |
| Succinic acid | 94.8 | 99.6 | 99.6 |  | - |
| N-Acetyl-L-glutamic acid | 88.6 | 99.2 | 99.2 |  | - |
| Citramalic acid | 72.8 | 81.9 | 81.7 |  | - |

^a^ Data represent mean scores obtained across multiple fixed collision energies (10, 20 and 40 V)
